# Supplementary material for: WordCommentsAnalyzer: A windows software tool for qualitative research
Source: F1000Res. 2018 Sep 4;7:536. Originally published 2018 May 3. [Version 2] doi: 10.12688/f1000research.14819.2 (PMC6137411; doi:10.12688/f1000research.14819.2)
Supplement: Supplementary file 3 [file f1000research-7-17614-s0002.tgz › b2a79496-4ada-47c4-9682-a99ce867836d.zip!/Eight Iranian Health Journals/Salmand_Iranian Journal of Ageing _26.docx]

© Salmand_Iranian Journal of Ageing . This work is licensed under CC BY-NC 4.0 (https://creativecommons.org/licenses/by-nc/4.0/).

Volume & Issue: دوره 6 - شماره 4

Title: بررسی فراوانی بیماری‌های مزمن در سالمندان شهر ایلام

http://salmandj.uswr.ac.ir/article-1-470-fa.html

اهداف: چاقی و افزایش وزن مهم‏‌ترین عوامل خطر قابل پیشگیری بیماری‌های قلبی عروقی و دیابت به ویژه در افراد مسن می‌‏باشند. پژوهش حاضر با هدف تعیین فراوانی بیماری‌های مزمن در سالمندان شهر ایلام انجام شده است. مواد و روش‌ها: در یک مطالعه توصیفی تحلیلی 121 نفر سالمند (%62/4 مرد) ساکن در شهر ایلام با میانگین سنی11/0±70/4 سال بصورت تصادفی و ازطریق نمونه‌گیری خوشه‌ای وارد مطالعه گردیدند. اندازه‌گیری قد، وزن و فشار خون از همه واحدهای مورد پژوهش، توسط یک پرستارمجرب صورت گرفت. یافته‌ها: میانگین شاخص توده بدنی  4/2±25/3  kg/m2 بود. بیش از نیمی از زنان %56/1 و %46/2 مردان، اضافه وزن یا چاقی داشتند. شیوع بیماری‌های قلبی و عروقی، فشار خون و دیابت طبق تشخیص پزشک به ترتیب %53 و %36/8 و %17/4 بود. ارتباط معنی‌داری بین فشارخون، درد قفسه سینه و گروه‌های سنی با شیوع بیماری قلبی وجود داشت (0/001>P). فشار خون سیستولیک در افراد بالای 75 سال به‌صورت معنی‌داری از فشار خون سالمندان 75-60 سال بالاتر بود (0/002>P)، در حالی که چنین ارتباطی در خصوص فشار خون دیاستولیک و شاخص توده بدنی مشاهده نگردید. بین سطح سواد و سابقه ابتلا به دیابت ارتباط معنی‌داری وجود داشت (0/000>P). همچنین سالمندانی که به طور مداوم و پیوسته تلویزیون نگاه می‌کردند بیش از سایرین به اضافه وزن و چاقی مبتلا بودند (0/05>P). نتیجه‌گیری: شیوع بیماری‌های مزمن در سالمندان مورد مطالعه با سن بالا، سواد و مدت زمان طولانی مشاهده تلویزیون مرتبط بود. شیوع چاقی و اضافه وزن در مطالعه حاضر در مقایسه با مطالعات صورت گرفته در سطح کشور همخوانی نزدیکی دارد. با این حال شیوع بیماری‌های قلبی و فشار خون بالاتر از سایر مطالعات کشوری به‌دست آمد. به منظور کاهش شیوع بیماری‌های مزمن در سالمندان برنامه‌های غربالگری و آموزش بهداشت پیشنهاد می‌گردد.

Title: شیوع تداخلات دارویی بالقوه در بین بیماران سالمند بستری در بخش مراقبت‌های ویژه

http://salmandj.uswr.ac.ir/article-1-471-fa.html

اهداف: مطالعه حاضر با هدف بررسی میزان تداخلات دارویی بالقوه در بین بیماران سالمند بستری در بخش مراقبت‌های ویژه بیمارستان شهید باهنر کرمان طراحی و انجام گردید. مواد و روش‌ها: در مطالعه مقطعی حاضر نسخ 24 ساعت اول تمامی بیماران سالمند بستری در بخش مراقبت های ویژه از تاریخ 1/1/1388 تا 1/1/1389 از نظر تعداد و نوع تداخلات دارویی و عواملی از قبیل تعداد داروهای دریافتی، سن، جنس، مدت زمان بستری در بخش مراقبت‌های ویژه و تعداد پزشکان تجویز کننده دارو مورد بررسی قرار گرفت. به منظور تعیین تعداد و نوع تداخلات داروی از کتاب مرجع تداخلات دارویی Facts استفاده گردید و به منظور تجزیه و تحلیل آماری داده ها در نرم افزار آماری SPSS 18 متناسب با اهداف پژوهش از آمار توصیفی، آزمون های آماری همبستگی پیرسون، تی مستقل و آنالیز واریانس استفاده شد. یافته‌ها: در مجموع 77 قلم داروی متفاوت در نسخ بیماران مشخص گردید و در کل 394 مورد تجویز دارو در نسخه ها یافت شد که به طور متوسط سهم هر بیمار، 5/62 دارو (1/5=SD) بود. در مجموع بررسی داروهایی که بیماران بخش مراقبت‌های ویژه در 24 ساعت اول دریافت کرده بودند، 108 مورد تداخل دارویی یافت گردید. از نظر نوع تداخل تداخلات تاخیری (Delayed)، متوسط (Moderate) و امکان‌پذیر (Possible) بیشترین درصد تداخلات را به خود اختصاص داده بودند. چهار مورد تداخل دارویی ماژور رخ داده به ترتیب بین 1) سایمیتیدین و متادون 2) فورسماید و آمیکاسین 3) فنی توئین و دوپامین و 4) هپارین و آسپیرین رخ داده بود. نتایج آزمون همبستگی پیرسون نشان‌دهنده ارتباط معنادار بین تعداد تداخلات دارویی با تعداد داروی تجویز شده بود. نتایج آزمون آنالیز واریانس یک‌طرفه نیز نشان داد که میانگین تعداد تداخلات دارویی در بیمارانی که با بهبودی از بخش مرخص شده بودند بیشتر از سایر بیماران بود. نتیجه‌گیری: بیماران سالمند بستری در بخش مراقبت‌های ویژه در خطر بالایی از تداخلات دارویی قرار دارند که این امر مستلزم توجه بیشتر تیم درمانی به این موضوع می‌باشد.

Title: بررسی کیفیت زندگی سالمندان تحت پوشش مراکز بهداشتی-درمانی مناطق شهری استان مرکزی در سال 1389

http://salmandj.uswr.ac.ir/article-1-458-fa.html

اهداف: با افزایش رشد جمعیت سالمندان و تغییر ترکیب جمعیتی به سمت سالخوردگی از این پس نیازمند افزایش اطلاعات راجع به کیفیت زندگی مربوط به سلامت در این گروه سنی هستیم تا اگر چه نمی‌توانیم از این تغییر جمعیتی جلوگیری کنیم ولی با برنامه‌ریزی‌های مناسب کیفیت زندگی آن‌ها را افزایش داده و با ایجاد سالمندی فعال مشارکت آن‌ها را در فعالیت‌های خانواده و جامعه بیشتر کنیم و زمینه پیشرفت اقتصادی و رفاه کشورمان را فراهم کنیم. هدف از این مطالعه بررسی وضعیت کیفیت زندگی و عوامل تاثیرگذار بر آن در سالمندان 60 ساله و بالاتر ساکن مناطق شهری استان مرکزی بوده است. مواد و روش‌ها: این پژوهش از نوع مقطعی و توصیفی‌تحلیلی بود که بر روی 165 سالمند در مناطق شهری استان مرکزی انجام گرفته شد. ابزار جمع آوری اطلاعات شامل پرسشنامه عمومی و پرسشنامه 36 سوالی کیفیت زندگی بود. همچنین برای تجزیه و تحلیل نتایج از آزمون‌های آماری کای دو، فیشر، تی مستقل، ANOVA یک‌طرفه، رگرسیون و ضریب همبستگی پیرسون استفاده گردید. یافته‌ها: میانگین سنی نمونه ها 70/7 سال بود و 44/8 درصد نمونه‌ها مرد و 55/2 درصد زن بودند. میانگین امتیاز کلی کیفیت زندگی برابر 22±55/66، میانگین امتیاز بخش جسمی 21±51/32 و میانگین امتیاز بخش روانی 25±57/30 و میانگین امتیاز 8 بعد کیفیت زندگی به این شرح بود: بعد عملکرد جسمی 26±56/45، کاهش عملکرد به علت مشکلات جسمی 27±60/50، درد جسمی 26±38/51، سلامت کلی 26±45/53، نشاط 24±53/09، عملکرد اجتماعی 27±67/85، کاهش عملکرد به علت مشکلات روانی 45±57/14، سلامت روانی 25±62/88. همچنین نمرات کیفیت زندگی با جنس، شغل قبلی، سواد، فعالیت‌های اوقات فراغت، درآمد، ورزش فعلی، ابتلا به بیماری و مصرف دارو ارتباط معنی‌دار داشت (P<0/05). نتیجه‌گیری: نتایج این پژوهش نشان داد کیفیت زندگی سالمندان مورد بررسی در حد متوسط است و افراد از نظر سلامت روانی در مقایسه با سلامت جسمی وضعیت بهتری دارند. همچنین فاکتورهای مختلفی با کیفیت زندگی سالمندان در ارتباط هستند که بسیاری از آن‌ها مانند رفع تبعیض‌های جنسیتی، فراهم‌نمودن تسهیلات برای فعالیت‌های اوقات فراغت و ورزش این گروه سنی است با برنامه‌ریزی‌های جامع قابل اصلاح می‌باشند.

Title: بررسی میزان مصرف میوه و سبزیجات و عوامل مرتبط در سالمندان بستری در بیمارستان‌های شهر زاهدان

http://salmandj.uswr.ac.ir/article-1-472-fa.html

اهداف: جمعیت سالمندان در ایران رو به افزایش است. تغذیه یکی از عوامل مهم تعیین‌کننده سلامت سالمندان است. شواهد قابل توجهی وجود دارد که نشان می دهد مصرف کم میوه و سبزیجات مهم‌ترین عامل خطر برای بیماری‌های مزمن است. پژوهش حاضر با هدف بررسی میزان مصرف میوه و سبزیجات در سالمندان شهر زاهدان انجام گرفته است. مواد و روش‌ها: این مطالعه توصیفی دارای جنبه‌های تحلیلی بوده که بر روی 300 سالمند 60 سال و بالاتر بستری در بیمارستان‌های شهر زاهدان انجام گرفته است. روش نمونه‌گیری در دسترس، ابزار پژوهش پرسشنامه پژوهشگر ساخته بوده که روایی محتوی و نیز پایایی آن تعیین و تایید گردید. برای تحلیل داده‌ها از آمار توصیفی (فراوانی و درصد) و آمار استنباطی (آزمون کای دو) و با اطمینان بیش از %99 استفاده شد. یافته‌ها: نتایج پژوهش نشان داد میانگین سنی واحدهای مورد پژوهش 7/5±68/8 بود، واز نظر میزان تحصیلات 70درصد سالمندان مورد پژوهش بی‌سواد بودند. 69/6درصد ساکن شهر و 30/4درصد ساکن روستا بودند. از نظر وضعیت تاهل 69/7درصد واحدهای مورد مطالعه متاهل و 29/3درصد همسر فوت شده بودند. همچنین 97/7درصد واحد های مورد پژوهش کمتر از میزان توصیه شده از سبزیجات و 64 درصد کمتر از میزان توصیه شده از میوه‌ها استفاده می‌کرده‌اند. بین تحصیلات (P=0.001) و تأهل (P<0.001) و مصرف سبزیجات همچنین بین شهری و روستایی بودن (P<0.002) و تحصیلات (P<0.001)با مصرف میوه رابطه آماری معنادار وجود داشت و بین جنسیت و میزان مصرف میوه و سبزیجات رابطه آماری معناداری یافت نشد. مهمترین موانع عدم مصرف کافی میوه و سبزیجات عدم آگاهی، مشکلات اقتصادی و مشکلات دهانی و دندانی بوده است. نتیجه‌گیری: میزان مصرف میوه و سبزیجات در سالمندان مورد مطالعه بسیار کم است و با توجه به اهمیت نقش مصرف کافی میوه و سبزیجات در سلامت سالمندان برنامه‌ریزی آموزشی و مداخلات تغذیه‌ای برای این گروه ضروری است.

Title: بررسی میزان سوءرفتار توسط اعضای خانواده نسبت به سالمندان عضو کانون‌های جهاندیدگان شهر تهران

http://salmandj.uswr.ac.ir/article-1-468-fa.html

اهداف: پژوهش حاضر با هدف تعیین میزان سوءرفتار اعضای خانواده نسبت به سالمندان عضو کانون‌های جهاندیدگان شهر تهران و شناسایی عوامل مرتبط با آن در سال 1390 صورت گرفت. مواد و روش‌ها: پژوهش حاضر مطالعه‌ای مقطعی است که با مشارکت 379 سالمند (254 زن و 125 مرد) ساکن شهر تهران در محدوده سنی 65 سال و بالاتر عضو کانون های جهاندیدگان شهر تهران انجام شد. داده ها از طریق مصاحبه چهره به چهره با سالمندان در کانون های جهاندیدگان یا منازل سالمندان انجام شد. سالمندانی که بر اساس نسخه فارسی آزمون کوتاه وضعیت شناختی فاقد اختلال شناختی تشخیص داده شدند پس از کسب رضایت آگاهانه وارد پژوهش شدند. سپس، پرسشنامه بررسی سوءرفتار خانواده نسبت به سالمندان، مقیاس اندازه گیری فعالیت های روزمره زندگی و پرسشنامه محقق ساخته جمع آوری اطلاعات دموگرافیک و فردی برای آنان تکمیل گردید. داده ها پس از جمع اوری با استفاده از آماره های مجذور کای و تی تست تحلیل شدند. یافته‌ها: یافته‌های پژوهش نشان داد که 9/25 درصد از نمونه های پژوهش حداقل یکی از انواع سوءرفتار را تجربه کرده بودند. بیشترین میزان شیوع سوءرفتار مربوط به غفلت عاطفی (17/4درصد) و سوءرفتار روان شناختی (17/2درصد)، و کمترین آن مربوط به طردشدگی (3/7درصد) و سوءرفتار جسمی (4/7درصد) بود. بین میزان سوءرفتار و متغیرهای جنس (0/0001>P)، بیمه درمانی (0/04>P)، شغل (0/0001>P)، کفایت منابع مالی (0/0001>P)، وضعیت سوءمصرف مواد توسط سالمند (0/017>P)، و میانگین متغیرهای سن (0/026>P)، و تعداد فرزندان (0/03>P)، وضعیت عملکردی (0/0001>P) و تعداد بستری مجدد (0/0001>P) بین دو گروه سالمندان تحت سوءرفتار قرار گرفته و تحت سوء رفتار قرار نگرفته تفاوت معناداری وجود دارد. نتیجه‌گیری: با توجه به میزان بالای سوءرفتارنسبت به سالمندان، توجه ویژه و اتخاذ تدابیری به منظور پیشگیری از تبعات بلند مدت آن مورد نیاز است.

Title: بررسی تاثیر کتاب‌درمانی برمیزان توانایی‌های خودمدیریتی سالمندان

http://salmandj.uswr.ac.ir/article-1-467-fa.html

اهداف: هدف اصلی این مطالعه، تعیین تاثیر کتاب درمانی بر میزان توانایی های خودمدیریتی سالمندان بود. مواد و روش‌ها: پژوهش حاضر یک مطالعه نیمه‌تجربی است. جامعه پژوهش شامل کلیه سالمندان 65 سال به بالای عضو کانون جهاندیدگان منطقه چهار شهر تهران بودند. نمونه شامل 60 نفر سالمند بود که با توجه به معیارهای ورود و خروج انتخاب شده بودند. اعضای نمونه با استفاده از جدول اعداد تصادفی در دو گروه مداخله (n=30) و گروه شاهد (n=30) قرارگرفتند. مداخله شامل پنج جلسه کتاب درمانی در طول ده هفته بود که برای گروه مداخله انجام شد. توانایی خودمدیریتی قبل و بعد از مداخله در هر دو گروه اندازه‌گیری شد. ابزار پژوهش، پرسش‌نامه SMSA-30 بود (0/8=α). برای تجزیه و تحلیل داده‌ها از نسخه 18 نرم‌افزار SPSS استفاده شد. داده‌ها با کمک آزمون‌های آماری کای اسکوئر، کلموگروف اسمیرنوف، دقیق فیشر، تی زوجی و تی مستقل تجزیه و تحلیل شدند. یافته‌ها: میانگین نمرات توانایی خودمدیریتی قبل از مداخله، در گروه آزمون 70/77 و در گروه شاهد 68/46 بود. پس از مداخله میانگین نمره توانایی خودمدیریتی در گروه آزمون 73/25 و در گروه شاهد 69/53 بود. نتایج در سطح %95 از لحاظ آماری معنادار بود. یافته های حاصل از پژوهش نشان می‌دهد که پس از انجام مداخله، نمره کلی توانایی‌های خودمدیریتی پس مداخله در دو گروه آزمون و شاهد، تفاوت معناداری از لحاظ آماری دارد (P=0/03). همچنین با توجه به یافته های مطالعه حاضر مشخص می شود که نمرات گروه آزمون در خرده مقیاس های سرمایه گذاری رفتاری، تنوع رفتاری و مثبت اندیشی بطور معناداری بالاتر از گروه شاهد است که نشان دهنده بهبود این خرده مقیاس‌ها در گروه آزمون نسبت به گروه شاهد است (P<0/05) که تاثیر مثبت مداخله را نشان می‌دهد. نتیجه گیری: یافته‌های پژوهش حاضر، ضمن حمایت از فرضیه تحقیق نشان داد که کتاب درمانی، موجب بهبود شاخص‌های توانایی‌های خودمدیریتی می‌شود که این امر بدان معنی است که این مداخله، می‌تواند سبب بهبود محسوس و معنادار توانایی‌های خودمدیریتی سالمندان و در نتیجه بهبود کیفیت زندگی آن‌ها شود. لذا انجام مداخلات غیرتهاجمی نظیر کتاب درمانی و سایر درمان‌های شناختی-رفتاری، توسط پرستاران می‌تواند ضمن بهبود توانایی‌های خودمدیریتی سالمندان، زمینه را جهت ارتقای کیفیت زندگی و حضور فعال‌تر آن‌ها در جامعه فراهم کند، ضمنا این مطالعه یک حوزه جدید جهت پژوهش‌ها و خدمات پرستاری معرفی کرده است.

Title: بررسی درک گفتار با فشردگی زمانی در سالمندان

http://salmandj.uswr.ac.ir/article-1-469-fa.html

اهداف: از جنبه تاریخی، اغلب مطالعات انجام شده در حیطه سالمندی و شنوایی بر اختلالات درک گفتار در سالمندان تمرکز دارند. بنابر مطالعات، اغلب اختلالات بازشناسی گفتار در سالمندان از نقص در پردازش زمانی شنوایی ناشی می شود. در مطالعه حاضر، توانایی سالمندان در بازشناسی گفتار با فشردگی زمانی مورد بررسی قرار گرفت. مواد و روش‌ها: نسخه فارسی آزمون گفتار با فشردگی زمانی با 6 فهرست کلمات تک هجایی در سه نسبت فشردگی صفر، 40 و 60 درصد روی 36 جوان 18 تا 30 سال و 32 سالمند 60 تا 80 سال از دو جنس با شنوایی هنجار در هر گوش، انجام شد. یافته‌ها: در هر دو گروه، بین امتیاز بازشناسی واژه ها در سه نسبت فشردگی، تفاوت معناداری وجود داشت. بین جوانان و سالمندان در هر سه نسبت فشردگی، تفاوت معناداری مشاهده شد (0/0001>P). بین دو گوش و دو جنس در نسبت های فشردگی مختلف، اختلاف معناداری مشاهده نگردید. نتیجه‌گیری: با افزایش سرعت گفتار، امتیاز بازشناسی کلمات به میزان قابل ملاحظه ای کاهش می یابد و سالمندی به میزان چشمگیری بر توانایی پردازش محرکات گفتاری سریع، تاثیر دارد.

Title: هزینه‌های درمانی و بیماری‌ها در سالمندان تحت پوشش سازمان بیمه خدمات درمانی استان تهران (87-1386)

http://salmandj.uswr.ac.ir/article-1-459-fa.html

اهداف: امروزه پدیده سالمندی و مسائل و مشکلات مربوط به آن موضوع بسیار مهمی می‌باشد که مورد توجه اندیشمندان علوم مختلف قرار گرفته است و پرداختن به این مسئله از جنبه‌های مختلف، اهمیت فوق‌العاده‌ای پیدا کرده است. هر دوره‌ای از عمر بشر مشکلات خاص خود را دارد. در این مقطع جوامع سالخورده و سالمندان با مسائل و مشکلات خاص دوران سالمندی از قبیل مشکلات اقتصادی، اجتماعی، فرهنگی، بهداشتی، درمانی و ... مواجه هستند. بنابراین بررسی بیماری‌های سالمندان و هزینه‌های درمانی بسیار بالای آن‌ها به عنوان یک موضوع محوری ضرورت دارد. تحقیق حاضر سعی دارد ضمن بررسی بیماری‌هائی که در افراد سالمند تحت پوشش سازمان بیمه خدمات درمانی استان تهران بروز می‌کند به بررسی هزینه‌های درمانی این بیماری‌ها بپردازد. مواد و روش‌ها: پژوهش حاضر یک تحقیق توصیفی تحلیلی است که بصورت کتابخانه‌ای صورت گرفته است. در این مطالعه تعداد 183093 پرونده بستری مورد کنکاش قرار گرفته و با بررسی اطلاعات هزینه‌ای موجود در اداره کل بیمه خدمات درمانی استان تهران و بیماری‌های مرتبط با آن، هزینه‌ها، بیماری‌ها و اقدامات درمانی این گروه مورد بررسی و مطالعه قرار گرفته است. یافته‌ها: سالمندان (گروه سنی بالای 60 سال) علیرغم اینکه %31 مراجعات بستری به اداره کل را شامل می‌شوند لیکن %37 هزینه را بخود اختصاص داده است و موید هزینه‌های نسبتا بالای این گروه سنی از بیمه شدگان می‌باشد. به عبارتی میانگین هزینه هر بار بستری در گروه سنی بالای 60 سال بیشتراز مقدار میانگین کل و معادل 4634384 ریال می‌باشد، این در حالی است که میانگین هزینه در سایر گروه‌های سنی بجز گروه 45 تا 59 سال بسیار کمتراز میانگین کل می‌باشد. مبلغ کل هزینه‌ها در گروه بالاتر از 60 سال از همه گروه‌های سنی بیشتر است. بررسی کدهای تشخیصی ICD در گروه سنی بالای 60 سال نشان می‌دهد که کدهای سایر بیماری‌های قلبی ریوی(I27)، آنژین قلبی(I20) و کاتاراکت(H25)، بیماری های ایسکمیک قلبی (125)، و نارسایی قلبی (150)، شایع‌ترین تشخیص‌ها در بین سالمندان می‌باشد. شایع‌ترین کدهای جراحی (کالیفرنیا) در گروه سنی بالای 60 سال، مربوط به آنژیوگرافی عروق کرونر با میانگین هزینه 9116371 ریال، و سپس کاتاراکت می‌باشد. 15 درصد پرونده‌های گلوبال مربوط به گروه سنی سالمندان و از نظر هزینه‌ای معادل %23  پرونده‌ها مربوط به این گروه سنی می‌باشد. درآوردن عدسی داخل و خارج کپسولی گذاشتن لنز (کد 57 گلوبال)، فتق اینگوینال یک طرفه از هر نوع یا بدون اکسیزیون هیدروسل یا اسپرماتوسل بجز فتق اینگوینال مختنق (کد 28 گلوبال)، کوله سیستکتومی یا بدون کلانژیوگرامی یا اکسپلوریزلسیون مجاری صفراوی (کد 27 گلوبال)، رتبه‌های اول تا سوم شایع‌ترین اعمال گلوبال سالمندان را تشکیل می‌دهند. به‌عبارتی بررسی الگوی بیماری در این گروه سنی بیانگر وجود بیماری‌های قلبی عروقی در راس جدول بیماری‌های شایع می‌باشد که این بیماری‌ها از بار هزینه‌ای نسبتا بالایی برخوردار می‌باشند. آزمون همبستگی پیرسون بین سن و مدت اقامت و هزینه‌های درمان همبستگی مثبت نشان می‌دهد.  نتیجه‌گیری: بیمه‌شدگان اداره کل در آینده نزدیک، دارای رشد روزافزون در جمعیت بالای 60 سال خواهند بود. بالا بودن هزینه‌های درمانی گروه‌های سنی بالای 60 سال باعث افزایش هزینه‌های درمانی سازمان‌های بیمه‌گر خواهد شد. روند پیرشدن جمعیت، موید این نکته است که سازمان‌های بیمه‌گر می‌بایست به‌شدت به فکر آمادگی لازم برای مدیریت بهینه منابع باشند.
